# VAMOS-Cam: Versatile affordable modular stackable camera system

**DOI:** 10.1016/j.ohx.2026.e00796

**Published:** 2026-05-24

**Authors:** Carsten Schmerbeck, Michael Heizmann

**Affiliations:** Karlsruhe Institute of Technology (KIT), Institute of Industrial Information Technology (IIIT), Hertzstraße 16, (06.35), 76187 Karlsruhe, Germany

**Keywords:** Machine vision, Cameras, Calibration

## Abstract

In this article, we propose a versatile and modular camera system designed for a wide range of research applications, with a particular focus on multi-camera and camera array setups. Current camera setups—whether single, stereo, or array configurations—are rarely available off-the-shelf and typically require custom mechanical assemblies. The proposed system addresses this limitation by providing a building-block-style catalog of components that can be easily fabricated and assembled. It leverages modern consumer-grade hardware, which has significantly improved in recent years, offering affordable alternatives to industrial camera systems without sacrificing functionality. The modular design enhances reproducibility and flexibility and enables research into advanced imaging methods, including generic intrinsic and extrinsic camera calibration.

## Graphical abstract

**Figure:**
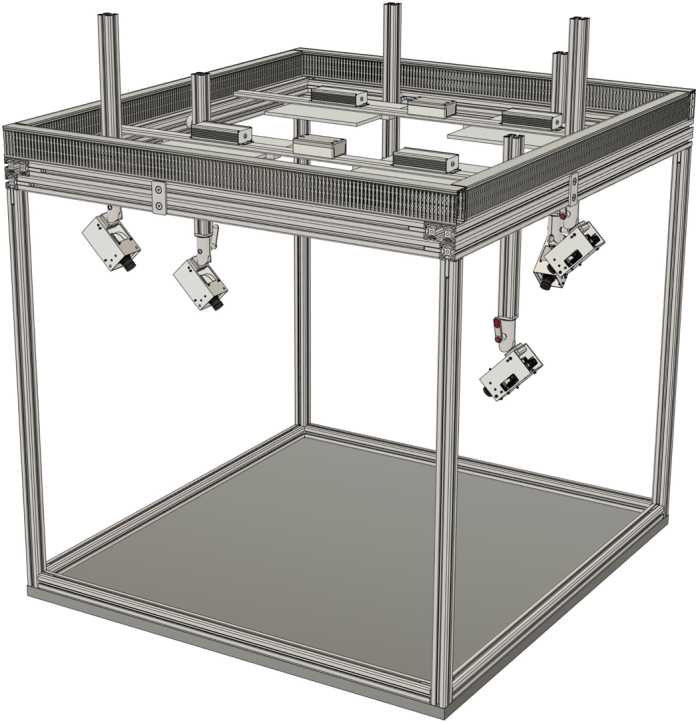

## Specifications table

**Table:** 

Hardware name	Versatile Affordable Modular Stackable Camera System

Subject area	•Engineering and material science • Educational tools and open source alternatives to existing infrastructure

Hardware type	•Imaging tools • Measuring physical properties and in-lab sensors • Electrical engineering and computer science

Closest commercial analog	The proposed system is a low-cost modular replacement for industrial cameras and machine vision systems. There are commercial analogs for individual components (e.g. cameras), but for the entire system, there is no commercial analog available.

Open source license	Creative Commons Attribution-Non Commercial 4.0 International (CC BY-NC 4.0)

Cost of hardware	Approx. 13 000 EUR
Source file repository	https://doi.org/10.17632/75x3v4jyr2.3

## 1 Hardware in context

Computer vision is a topic of growing interest for various fields of research. It has evolved from a niche discipline into a foundational technology for research and development across a vast spectrum of scientific and engineering domains. At the core of these use cases lies the need for precise, affordable and versatile image acquisition, which often involves multiple cameras [1].

Depending on the scientific question, researchers typically require one of three hardware configurations: individual cameras [2], [3], stereo cameras [4], [5] and grid-like camera arrays [6], [7]. Often they require overlapping fields of view [8].

As such systems are not available off-the-shelf [9], [10], researchers resort to designing their own setups [11] which forces them to reinvent mechanical fixtures, power distribution, and synchronization circuits, incurring unnecessary engineering effort and hindering reproducibility [12].

Despite the growing availability of affordable sensor modules, no open-source platform currently offers a truly modular, stackable, and network-scalable architecture that can be reconfigured on-the-fly for arbitrary single, stereo, or array layouts whilst providing the infrastructure for generic intrinsic [13] and extrinsic camera calibration [14]. For these reasons, we introduce the VAMOS-Cam: **V**ersatile **A**ffordable **Mo**dular **S**tackable **Cam**era System.

This work aims to reduce this effort significantly by providing a building-block-like catalog of components that can be easily manufactured and assembled. Furthermore, it increases reproducibility whilst allowing for easy modifications to fit application-specific requirements [12].

## 2 Hardware description

This camera system is intended for all applications where multiple low-cost cameras are needed. The cameras are stackable in all directions (Fig. 3), allowing for the generation of stereo images or the recording of light fields [6], [7].

Moreover, an automated intrinsic calibration setup is provided, which is suited for generic camera calibration. This model-free method of calibration is characterized by treating each pixel individually without any geometric constraints regarding the camera optics and therefore allowing for very precise calibration [13], [15], [16]. However, other calibration methods are suited as well.

**Fig. 1:**
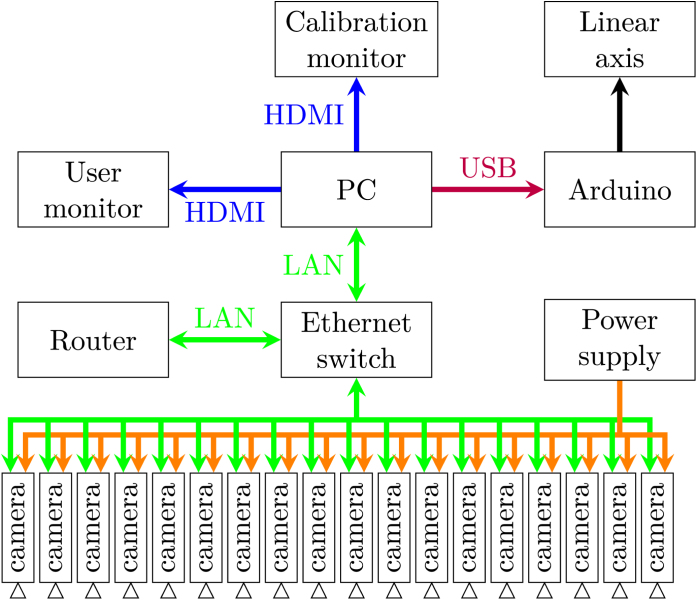
System overview.

The number of cameras can be varied and the hardware enables the usage of up to 253 cameras in the same network. However, this setup is limited in recording speed by the Ethernet connection of the used PC and switch. A system overview is illustrated in Fig. 1. The design can be broken down into individual building blocks that shall be explained in this section.

### 2.1 Scope and intended usage

This project is not meant to be a consumer-grade or market-ready product, but a platform targeted to simplify the deployment and development of such a camera system in a research context and should serve as a starting point for further designs. Its planning includes many design choices that can be changed freely in future implementations to suit one’s needs and requirements. Furthermore, we do not prescribe any downstream software or infrastructure in order to keep the system’s flexibility beyond its original intended usage of wiring harness recognition with generic camera calibration [13], [14]. The camera system is suited for a multitude of indoor applications that require uncompressed image recordings of cameras in various individual or array arrangements.

**Fig. 2:**
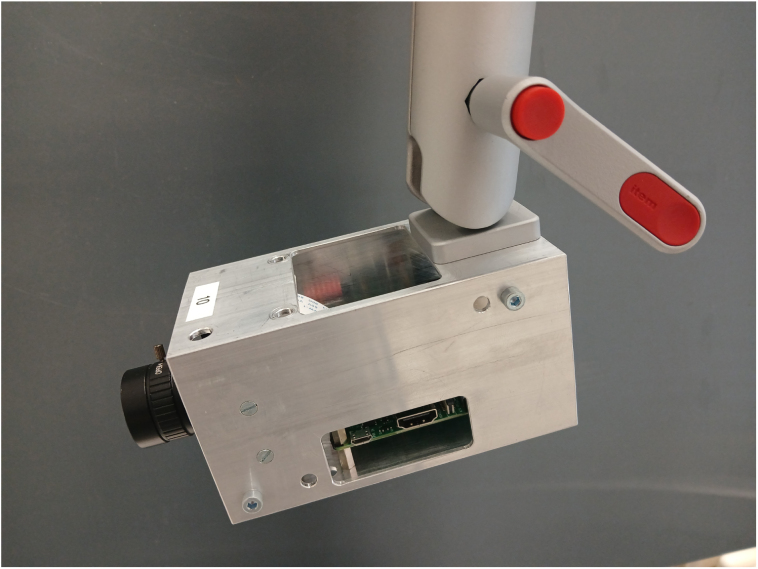
Custom-built camera module with ball-joint swivel mount.

**Fig. 3:**
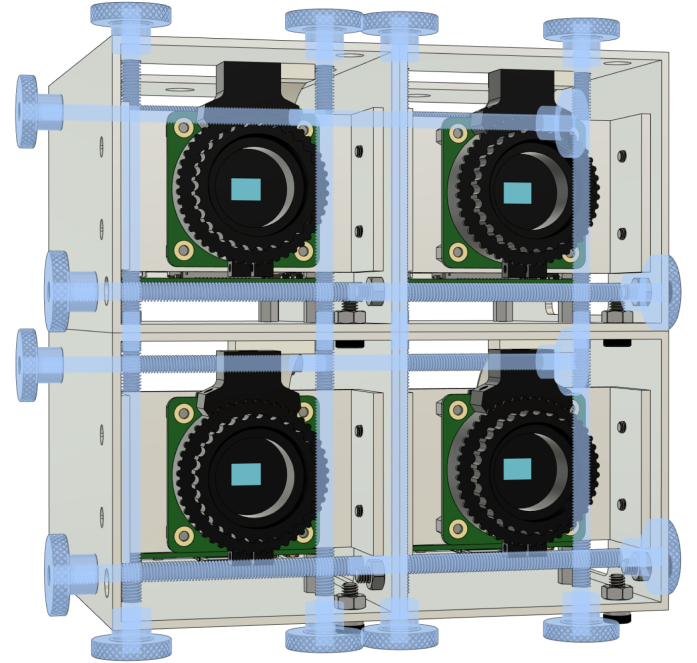
Stacking of cameras using all-thread and knurled nuts.

### 2.2 Camera modules

The camera modules (Fig. 2) are based on Raspberry Pi High Quality Camera Modules [17], offering a resolution of 12.3 megapixels. For more details, see Table 1. Each camera module contains a Raspberry Pi 3 which sends the images to a central PC. We used this popular microcomputer as it is very common among researchers and there is a large community and vast availability of open-source solutions, especially in the context of computer vision [3], [12], [18]. Although not implemented in this camera system (as this is very application-specific) it is easily possible to perform certain steps of preprocessing or even the full computation on the Raspberry Pi modules themselves, thereby reducing the processing load on the central PC — a feature that standard cameras typically lack [3], [12].

Each Raspberry Pi is equipped with a mezzanine board, providing a regulated power supply to it, as well as handling the hardware triggering of a recording if necessary. However, this module could be operated on its own, with minimal surrounding infrastructure (Power and Ethernet).

**Table 1:** Technical data of the camera module [17] and lens [19].

Spec	Value
Sensor	Sony IMX477R
Resolution	4056×3040, 12.3MP
Pixel size	1.55µm×1.55µm
Lens mount	CS
Lens focal length	6mm
Lens field angle	63°
Aperture	F1.2

Each camera module is equipped with a swivel head to rotate the camera freely, as well as a system to stack cameras arbitrarily. To show the usage of two three by three arrays of cameras, we manufactured 18 units.

### 2.3 Trigger system

A recording can be started by one of three mechanisms:

• The recording can be requested via the Ethernet connection of the Raspberry Pi. As described in the hardware documentation, it provides a Flask server that enables the user to remotely change camera settings, such as gain and exposure time. Furthermore, it replies with the recorded image.
• Alternatively, the hardware allows for triggering of a recording via a dedicated hardware signal, optically isolated from the Raspberry Pi. This allows for triggering from external sources, such as hardware that should be recorded at a certain time instance. There is an option to generate such a trigger signal simultaneously for all cameras via a dedicated driver board.
• Alternatively, the software supports the direct streaming of a video with reduced resolution to a browser. This option is well-suited for long-term observations or surveillance.

### 2.4 Switching cabinet

**Fig. 4:**
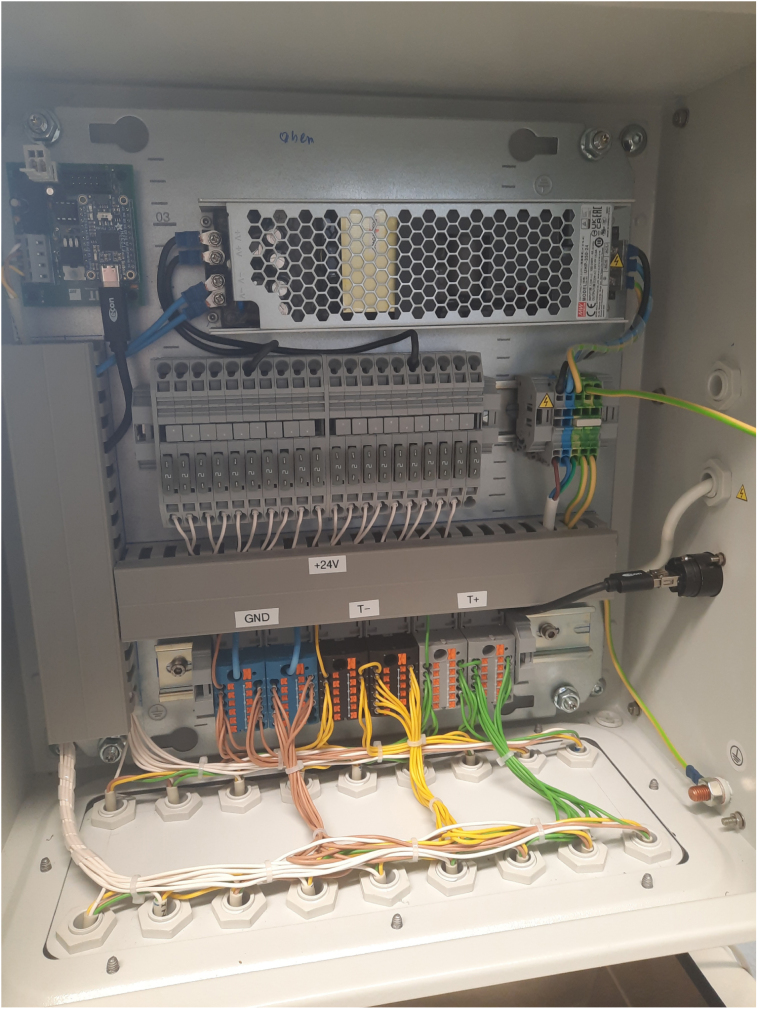
Switching cabinet interior.

To supply the infrastructure needed for the camera system to work, there is a switching cabinet housing a power supply, distribution and fuses. A dedicated driver PCB enables hardware synchronization of all cameras (Section 2.2). It is depicted in Fig. 4. Furthermore, there is an Ethernet switch attached to it to connect all of the cameras.

### 2.5 Intrinsic calibration setup

**Fig. 5:**
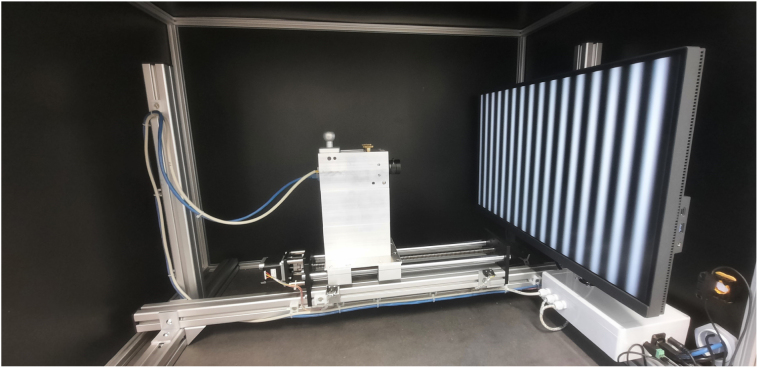
Intrinsic calibration setup.

To be used with approaches of generic camera calibration, we devised a setup with a monitor as an active target, as depicted in Fig. 5. We successfully showed that this system, together with implementations by Uhlig [13], is capable of successfully calibrating the utilized cameras generically with great accuracy [14], [20], [21].

The setup consists of a rigid frame constructed from 40 mm aluminum extrusion profiles that supports both the calibration monitor and the camera linear axis. A 4K BenQ Monitor is mounted via its VESA100 interface, while the camera is positioned on a motorized linear axis with 400 mm travel driven by a NEMA17 stepper motor. This enables automatic capture of fringe projection patterns at multiple distances between the monitor and the camera.

The optical enclosure, built from 20 mm aluminum profiles with black-coated cover plates, shields the calibration from stray light. Power is provided by a 24 V power supply, with a PoStep25-256 stepper driver and Arduino Uno controlling the linear axis. Safety is ensured by a door interlock and an emergency stop switch.

### 2.6 Extrinsic calibration setup

**Fig. 6:**
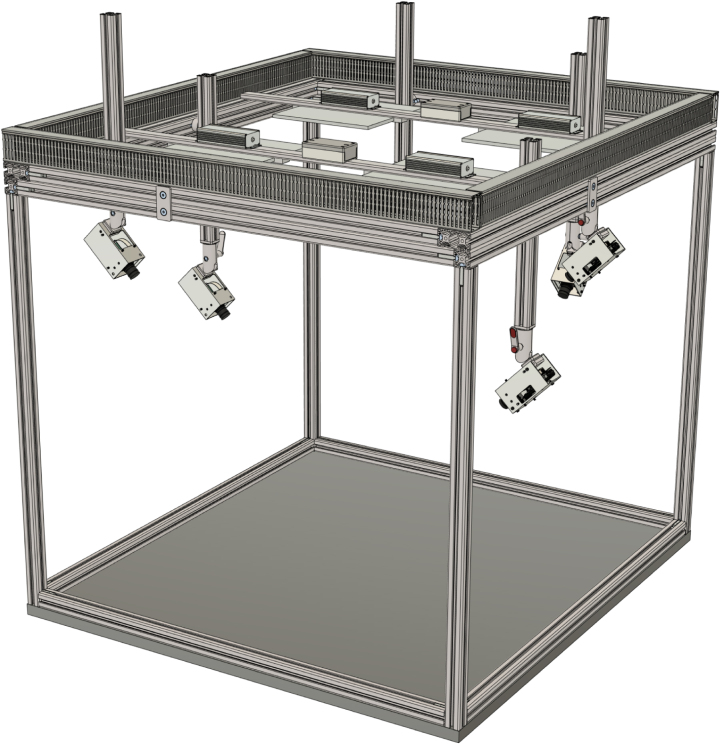
Extrinsic calibration setup rendering.

**Fig. 7:**
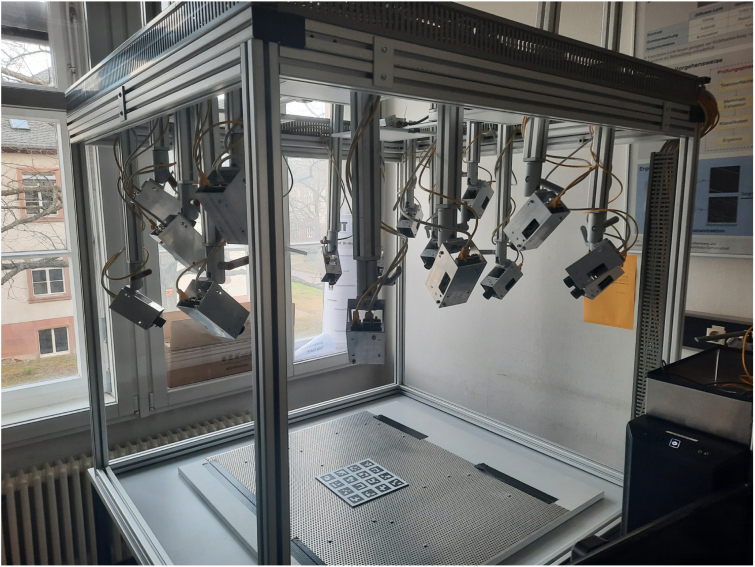
Extrinsic calibration setup.

To position the cameras rather freely around a certain working volume, we constructed a carrier frame, which enables the free placement and adjustment of up to 18 camera modules (Fig. 6, Fig. 7). It resembles a robot cell, where the top part of the camera system can be mounted atop, referencing one of the prior usages [14]. It is equipped with a set of lights to evenly illuminate the workspace. The lighting system provides uniform 3000 lx illumination via four LED panels mounted on the top crossbars.

The frame provides the infrastructure for determining the relative poses of all cameras within the measurement volume (extrinsic calibration). Calibration targets with ArUco markers are placed at predefined positions in the scene to enable robust pose estimation. They can either be positioned by a robot arm carrying the calibration board, as demonstrated in Zürn et al. [14], or by a manual variant using a perforated sheet with alignment pins [21].

### 2.7 Example images

**Fig. 8:**
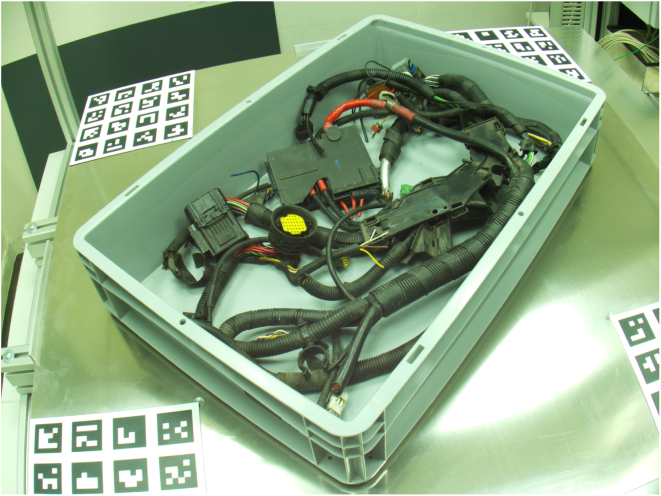
Example image.

**Fig. 9:**
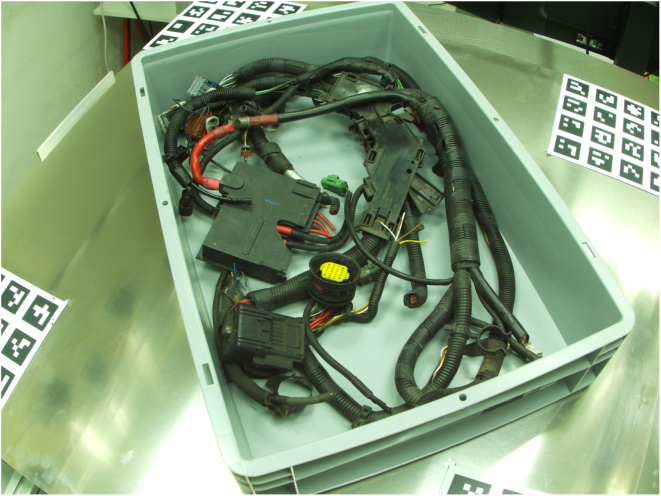
Example image.

Fig. 8, Fig. 9 are two examples for camera recordings. They show an automotive wiring harness in a box [14]. They both have a size of 4056 × 3040 pixels.

### 2.8 Advantages over pre-existing methods

In the past, when researchers were in need of a camera system for an application, they often resorted to designing a task-specific system from scratch. Table 2 lists several representative examples. This work aims to simplify the process by providing an open-source and low-cost solution that can be easily deployed and adapted to specific needs. Thus, the duplication of development effort across research groups can be avoided. Furthermore, this system can be reused easily, which is advantageous for rather short-term projects.

**Table 2:** Comparison to other multi-camera setups.

Source	[6] 2018	[22] 2010	[23] 2002	[24] 2004	[25] 2022	Ours 2026
Number of cameras	100	11	5–6	4 and 2	8	18

Sensors	5 MP OV5647 (Raspberry Pi Camera)	Unknown type	0.3 MP PRYO 1394 or 0.3 MP Sony DFW-V500	0.36 MP MT9V022 (AVT Guppy F-036) and 0.45 MP IXC415 (AVT Stingray F-046)	6.3 MP IMX178 (MV-CA060-10GM)	12.3 MP IMX477R (Raspberry Pi HQ Camera)

Arrangement	Grid	Individual	Individual	Individual	Individual	Grid, line or individual

Connection	WiFi & 100 Mbit s^-1^ Ethernet	1 Gbit s^-1^ Ethernet	1 Gbit s^-1^ Ethernet	IEEE 1394 a/b	1 Gbit s^-1^ Ethernet	1 Gbit s^-1^ Ethernet

Application	Light-field measurements	Fly tracking	Person localization	General	Tube profile measurement	General

Versatility	Camera placement strongly constrained by fixed grid	Application-specific	Application-specific	System size strongly limited due to IEEE 1394	Application-specific	High

Remarks	Signal interference for CSI connection	Real-time tracking	Synchronized, low-cost	Synchronized	Very comparable setup	–

### 2.9 Comparison with commercial camera systems

The major advantages over commercial systems are the system being fully open-source and very affordable. Commercial systems often do not allow for such large versatility. In addition, the modular design enables researchers to easily scale the number of cameras and adapt their spatial arrangement to different experimental needs. The hardware components are widely available consumer products, which makes replacement and maintenance straightforward. Furthermore, the open availability of design files, software, and calibration procedures ensures reproducibility and facilitates collaborative development. The system therefore combines low-cost, versatility, and transparency, making it highly suitable for a broad range of research applications.

**Table 3:** Design files that include all documents required to fabricate and assemble the VAMOS-Cam System.

Design filename	File type	Open source license	Location of the file
System design, building instructions and user manual	Document (.pdf)	CC BY-NC 4.0	https://doi.org/10.17632/75x3v4jyr2.3

KiCad library	KiCad 8 (.pretty)	CC BY-NC 4.0	https://doi.org/10.17632/75x3v4jyr2.3

Extrinsic setup schematics	KiCad 8 (.kicad_sch)	CC BY-NC 4.0	https://doi.org/10.17632/75x3v4jyr2.3

Intrinsic setup schematics	KiCad 8 (.kicad_sch)	CC BY-NC 4.0	https://doi.org/10.17632/75x3v4jyr2.3

Mezzanine PCB	KiCad 8 (.kicad_sch & .kicad_pcb)	CC BY-NC 4.0	https://doi.org/10.17632/75x3v4jyr2.3

Driver PCB	KiCad 8 (.kicad_sch & .kicad_pcb)	CC BY-NC 4.0	https://doi.org/10.17632/75x3v4jyr2.3

Intrinsic setup CAD	CAD (.step)	CC BY-NC 4.0	https://doi.org/10.17632/75x3v4jyr2.3
Cabinet CAD	CAD (.step)	CC BY-NC 4.0	https://doi.org/10.17632/75x3v4jyr2.3
Extrinsic setup CAD	CAD (.step)	CC BY-NC 4.0	https://doi.org/10.17632/75x3v4jyr2.3

## 3 Design files summary

The PDF file contains detailed instructions on the manufacturing process, required tools and gives insight into the design of the components. Furthermore it states instructions on operation, as well as safety considerations. All schematics (PCB and non-PCB) were drawn in KiCad 8, which was also used to keep track of parts lists. The files designated with ‘PCB’ include a printed circuit board design. The CAD files illustrate all mechanical components of the setup and are supplemented by detailed mechanical drawings, by which the components that require manufacturing can be produced.

## 4 Bill of materials summary

This list contains approximate costs of the hardware. Please note, that this may be subject to significant changes. The costs do not include the working hours for manufacturing and assembly, tooling, machine maintenance or wear (see Table 4).

**Table 4:** Bill of materials.

Designator	Component	Number	Cost per unit - currency	Total cost - currency	Source of materials	Material type
Mezzanine PCB	Circuit Board	18	10.25 €	184.50 €	www.multi-circuit-boards.eu	Composite

Driver PCB	Circuit Board	1	13.18 €	13.18 €	www.multi-circuit-boards.eu	Composite

Camera electronics	Components	18	204.46 €	3680.41 €	Various^a^	Other

Calibration boards	ArUco	10	30.41 €	304.10 €	www.hannes-beschriftungen.de	Composite

Intrinsic Calibration setup	Components	1	1741.11 €	1741.11 €	Various^a^	Other

Extrinsic Calibration setup	Components	1	2538.89 €	2538.89 €	Various^a^	Other

Camera case	Camera case	18	1.45 €	26.22 €	www.eisen-schmitt.de	Metal

Switching cabinet	Switching cabinet	1	799.28 €	799.28 €	Various^a^	Other

PC	PC	1	3948.91 €	3948.91 €	www.net-factory.de	Other

a There are detailed descriptions of the used materials and components as well as their manufacturers in the complete documentation (see Table 3). Some generic components such as screws and washers are not designated with specific manufacturers.

## 5 Build instructions

Detailed step-by-step instructions on the assembly process can be found in the supplementary instructions document. Design decisions and design alternatives are listed alongside schematics and mechanical drawings. The document also includes safety hints.

## 6 Operation instructions

There are detailed step-by-step instructions on the operation of the hardware in the supplementary document.

## 7 Validation and characterization

**Exemplary use case 1:** “Robotic Wiring Harness Bin Picking Solution using a Deep-Learning-based Spline Prediction and a Multi-Stereo Camera Setup” [14].

To show how this camera system might be applied to a machine vision task, we will outline this shortly for an exemplary case.

With increasing size, complexity and weight of wiring harnesses, their predominantly manual installation in vehicles is a very demanding task for human workers. Therefrom the need for an automated installation arises. One key step in the robotic handling of such flexible objects is a robust perception and localization of the wiring harness or its connectors.

**Fig. 10:**
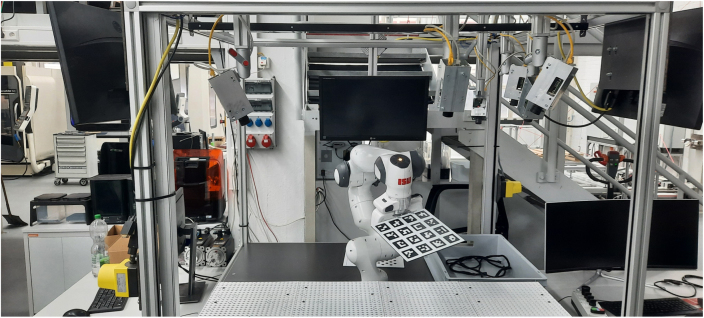
Example application [14].

Therefore, the VAMOS-Cam system was deployed to a robot cell which served as a rigid mounting structure for the cameras as shown in Fig. 10. Three of them were used for a bin-picking task.

**Fig. 11:**
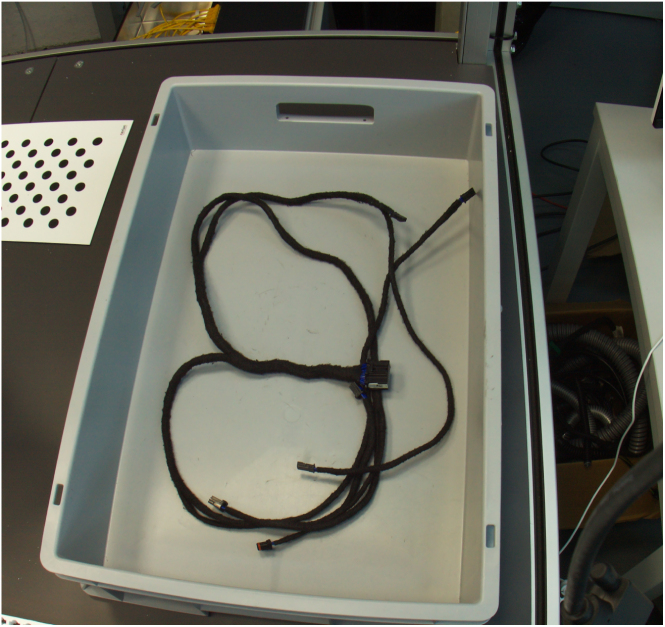
Wiring harness as seen from a VAMOS-Cam camera [14].

The generically-calibrated [13] VAMOS-Cam system is being used here to localize a grasping point, that has been detected by a deep learning model in the images (An example is depicted in Fig. 11). The robot then grasps the wiring harness and picks it up for further installation. The intrinsic calibration here is performed on the hardware described in this document based on a software implementation of generic camera calibration by [13]. The extrinsic calibration however is performed relative to the robot end effector, by mounting a ArUco marker board [18] at its end effector. This method used a novel ICP-based [26] method of extrinsic camera calibration that is suited for generically calibrated cameras [14].

Additionally, the calibration was evaluated for its accuracy by measuring fixed lengths on the ArUco marker boards, as well as evaluating the RMSE reprojection error for multiple point estimations. All of these errors lay in the millimeter range [14] which could be repeated in other setups as well [21]. Overall 50 grasping trials were performed, out of which 41 were successful. The errors occurred mostly due to collisions with the box, which was not implemented in this case. Other failures occurred due to robot kinematic singularities or a wrong spline prediction. The camera system did not cause any issues [14].

**Exemplary use case 2:** Simultaneous video recording for a demonstrator

To illustrate the procedure of a connector installation, a simultaneous video recording from multiple perspectives was required [27].

As the VAMOS-Cam system also allows for video streams to be directly output and recorded by other software, it was installed in a demonstrator robot cell. Here, three individual cameras were used. The video recording and arrangement was performed using OBS Studio [28]. The resulting video is available at [29].

Both of these use cases also proved, that the system is able to be disassembled and transported with limited effort, as both of these examples took place in different locations (US ISW Stuttgart and Fraunhofer IPA Stuttgart) than the original assembly (KIT IIIT Karlsruhe).

**Other use case examples 3–5:**

3. “Automotive Wire Harness Connector Installation Using Skill-Based Robotic Programming” [27]
  – Considerations on positional accuracy in connector installation.
4. “Anwendung generischer Kamerakalibrierung für optische Messsysteme in der Fertigungstechnik” [21]
  – Position estimation of a grasping point on a wiring harness.
  – Length measurement validation using ArUco markers.
  – Length measurement validation using the distance of two spheres.
5. “Optimal aperture settings for generic camera calibration” [20]
  – Influence of the cameras aperture setting on calibration accuracy.
  – Length measurement validation using the distance of two spheres.

These show that this system can be utilized for various tasks in machine vision [1]. Especially the limitations of the cameras, such as distortions, allow for both the usage of common, as well as the creation of new algorithms for calibration and measurements [14], [21]. Furthermore it can be used both for scientific evaluation of e.g. parameter studies [20] or for application-oriented use cases [14], [27].

### 7.1 Performance

The system allows for the recording of individual images with a high resolution of 4056 × 3040 pixels in a lossless format. The recording speed is limited by the Ethernet connection, resulting in an image acquisition and transport time for a single camera of 3.83 s in total (averaged over 10 measurements). However, this speed can be increased by utilizing a USB to Ethernet adapter (TP-Link UE300) which reduces this time to 1.76 s, averaged over 10 measurements. An image recording of all 18 cameras in the current setup takes 7.76 s, averaged over 10 measurements. All measured using python  from request until the images are stored on the PCs SSD.

**Fig. 12:**
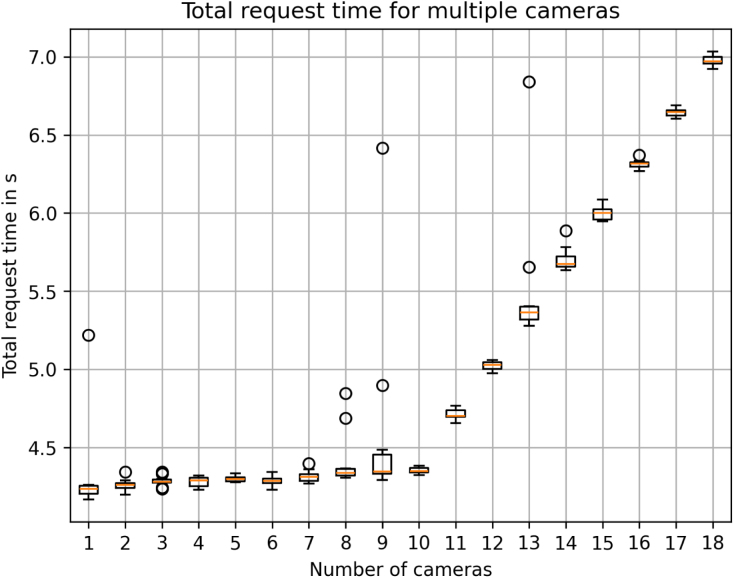
VAMOS-Cam capture performance for single images.

Fig. 12 depicts the total time for the recording of increasing numbers of cameras being used with 10 repetitions. One can see, that the system response time increases for larger number of cameras as the PCs Ethernet connection and switch reach the limit of their connection speed of 1 Gbit s^-1^.

During all experiments, we did not experience any loss of HTTP requests due to timeouts, as this period is set to 10 s to avoid such cases. One can observe in Fig. 12, that some of the requests take significantly longer as indicated by the marked outliers, presumably due to other activity on the network or non-real-time behavior of both the cameras and PC [30, Sec. I.4].

### 7.2 Management of recordings

An efficient and structured data management workflow is a prerequisite for the effective use of this setup, particularly for storing the generated images in a reusable and accessible manner [31, Sec. 3.1].

For this aspect, we employ a modular framework consisting of one Python script per experiment type. Each script documents the complete experimental procedure, including setup instructions (provided as comments), automated configuration of camera parameters, control of the recording process (e.g., for intrinsic calibration [14]), and subsequent data processing steps. In addition, the scripts define the directory structure used for data storage and manage associated metadata, such as scene configurations and timestamps. A template for these scripts is provided in the supplementary material.^1^ An example dataset generated using this framework is available in [32].

### 7.3 Video stream

For the video option, the system is able to record mjpg video streams (lossy) from all 18 cameras simultaneously at 30 fps with 1014 × 760 pixels using  . This resulted in a network data rate of about 460 Mbit s^-1^. This feature is mostly intended as a live preview in order to position and align a camera in a scene.

### 7.4 Calibration accuracy

The calibration accuracy performance was described in some previous publications, the following list gives some exemplary numbers for these specific setups.

• For the intrinsic calibration, reprojection errors in a range of up to 32 µm RMSE are typical for this calibration setup [14]. This has been proven by previous publications as well [13].
• Multiple experiments and setups show consistently low RMSE reprojection errors for the extrinsic calibration of below 4.5 mm [20]. The higher RMSE values in [14] of below 7.8 mm may be a result of the robot being used for calibration.
• Using the system for length measurements of the distance between two spheres, it performed with a standard deviation of 0.62 mm [20].

## 8 Outlook

To summarize, we created an affordable and versatile camera system, that can be easily configured and might be a helpful tool for all researchers with the need for a camera system in a research context. The platform is easily expandable, rugged, and due to the use of consumer hardware, easy to source. The entire system is relatively low-cost and completely open-source. The camera modules can be easily arranged for various stacks and arrays. From our experiments we can conclude that this camera system is suited for various tasks, such as taking measurements [20], [21] or industrial computer vision applications [14], [27]. Furthermore, we found some starting points for future research:

• One downside of the current setup is that due to limited availability, we used Raspberry Pi Model 3 boards. These only have a 100 Mbit s^-1^ Ethernet port. The usage of USB Ethernet dongles increased this number to roughly 200 Mbit s^-1^. Nevertheless, the preferable option would be to use Raspberry Pi Model 4 and newer, as they offer 1 Gbit s^-1^ ports. To utilize this additional capacity a faster switch and network adapter for the PC would be required.
• Secondly, it turns out that the physical trigger signal for image recordings is rarely used in the current setup and it incurs considerable hardware effort. In case the feature is not needed, it may as well be removed from the design in the future.
• The current power supply setup is not suited for very large scale deployment of many cameras. For the amount of 18 cameras shown here, it is reasonable. In case one would want to deploy this system in such a large scale, Power-over-Ethernet (PoE, namely by utilizing a Raspberry Pi PoE+ HAT [33]) could simplify the power supply setup for the case that a hardware trigger is not needed. However this solution would involve reconsidering many design choices made for the current setup.
• The video stream feature may be subject to future optimization and testing, as this is of interest for many fields of research [3].

## CRediT authorship contribution statement

**Carsten Schmerbeck:** Writing – review & editing, Writing – original draft, Visualization, Validation, Software, Methodology, Investigation, Formal analysis, Data curation, Conceptualization. **Michael Heizmann:** Writing – review & editing, Supervision, Resources, Project administration, Funding acquisition.

## Ethics statement

The authors declare that the work described has not involved experimentation on humans or animals.

## Funding

The authors would like to thank the Ministry of Science, Research and Arts of the Federal State of Baden-Württemberg, Germany for the financial support of the projects within the InnovationCampus Future Mobility.

## Declaration of competing interest

The authors declare the following financial interests/personal relationships which may be considered as potential competing interests: Carsten Schmerbeck reports financial support was provided by InnovationCampus Future Mobility. If there are other authors, they declare that they have no known competing financial interests or personal relationships that could have appeared to influence the work reported in this paper.

## Footnotes

1 https://doi.org/10.17632/75x3v4jyr2.3.
